# Dynamic Reconfiguration of Switchgrass Proteomes in Response to Rust (*Puccinia novopanici*) Infection

**DOI:** 10.3390/ijms241914630

**Published:** 2023-09-27

**Authors:** Nathan A. Palmer, Sophie Alvarez, Michael J. Naldrett, Anthony Muhle, Gautam Sarath, Serge J. Edmé, Satyanarayana Tatineni, Robert B. Mitchell, Gary Yuen

**Affiliations:** 1 Wheat, Sorghum, and Forage Research Unit, United States Department of Agriculture-Agricultural Research Service (USDA-ARS), Lincoln, NE 68583-0937, USA; nathan.palmer@usda.gov (N.A.P.); anthony.muhle@gmail.com (A.M.); serge.edme@usda.gov (S.J.E.); satyanarayana.tatineni@usda.gov (S.T.); rob.mitchell@usda.gov (R.B.M.); 2 Proteomics and Metabolomics Core Facility, Center for Biotechnology, University of Nebraska at Lincoln, Lincoln, NE 68588-0664, USA; salvarez@unl.edu (S.A.); mnaldrett@unl.edu (M.J.N.); 3 Department of Plant Pathology, University of Nebraska at Lincoln, Lincoln, NE 68583-0722, USA; gyuen1@unl.edu

**Keywords:** switchgrass, *Panicum virgatum*, rust, *Puccinia novopanici*, LC-MS/MS, plant defense, STRING analysis

## Abstract

Switchgrass (*Panicum virgatum* L.) can be infected by the rust pathogen (*Puccinia novopanici*) and results in lowering biomass yields and quality. Label-free quantitative proteomics was conducted on leaf extracts harvested from non-infected and infected plants from a susceptible cultivar (Summer) at 7, 11, and 18 days after inoculation (DAI) to follow the progression of disease and evaluate any plant compensatory mechanisms to infection. Some pustules were evident at 7 DAI, and their numbers increased with time. However, fungal DNA loads did not appreciably change over the course of this experiment in the infected plants. In total, 3830 proteins were identified at 1% false discovery rate, with 3632 mapped to the switchgrass proteome and 198 proteins mapped to different *Puccinia* proteomes. Across all comparisons, 1825 differentially accumulated switchgrass proteins were identified and subjected to a STRING analysis using Arabidopsis (*A. thaliana* L.) orthologs to deduce switchgrass cellular pathways impacted by rust infection. Proteins associated with plastid functions and primary metabolism were diminished in infected Summer plants at all harvest dates, whereas proteins associated with immunity, chaperone functions, and phenylpropanoid biosynthesis were significantly enriched. At 18 DAI, 1105 and 151 proteins were significantly enriched or diminished, respectively. Many of the enriched proteins were associated with mitigation of cellular stress and defense.

## 1. Introduction

Plants and their pathogens are constantly in a state of war and have evolved genetic mechanisms to overcome barriers posed by each other for effective survival [1]. The complexity of these interactions and the effects on plant yield can vary. Plants first need to perceive the presence of a pathogen before mounting defensive responses [2]. However, there are several nuances during pathogen perception that modulate the resultant cellular defensive responses [3,4]. Plants can also vary as to how effectively their tissues can withstand stress imposed by infection with a pathogen. This trait is associated with disease ‘tolerance’, a form of disease resistance in which a plant is able to sustain economically acceptable yields despite being infected by a pathogen [5].

Sustainable production of biomass from switchgrass is dependent on stable yields over several years and requires good local adaptation of the cultivars within their regions of production. Switchgrass is susceptible to several fungal and viral pathogens [6,7,8,9,10,11]. Significant losses in biomass and ethanol yields can occur from infestations with rust *Puccinia novopanici* (Pn), previously *Puccinia emaculata* [12,13,14], and there is uncertainty of yield stability of switchgrass monocultures under constant disease pressures. Within the context of the Central Great Plains of the US, breeding research has resulted in the release of the first bioenergy-specific cultivar named Liberty [15]. Liberty was derived from crosses initially made between plants of an upland cultivar, named Summer, and a lowland cultivar named Kanlow. Summer is significantly more susceptible to diseases than Kanlow [6,16]. Progeny populations derived by crossing Summer (♀) × Kanlow (♂; S × K) plants possess high biomass yields, with intermediate resistance to infection [6]. There is considerable variation in rust resistance among switchgrass lines [16,17], and a strong environmental effect on the severity of rust symptoms [18].

Switchgrass transcriptomes are changed within a short time after rust infection, with genes encoding kinases being upregulated within 1 h of infection, followed by the upregulation of genes encoding oxidoreductases, cofactor binding proteins, and those involved in defense responses [13], although no proteomic changes were presented. It can be anticipated that more rapid changes at the transcriptional level will usually take longer to be manifested at the protein level. It is also plausible that fast transient increases in proteins such as transcription factors and signaling proteins may be more difficult to quantitate. Additionally, proteins are subject to a wide range of post-translational modifications (PTMs) and turnover dynamics that can affect function, cellular location, and abundances. These protein-specific cellular mechanisms also impact proteomic analyses. Physiological outcomes at longer durations of rust infections on switchgrass have not been reported but can provide data on how plants respond to persistent stress induced by the fungal pathogen. These longer-term responses will have a significant impact on biomass quality since they determine how effectively plant metabolism copes with energy required for growth versus defense.

Proteomic studies have been performed on switchgrass under natural and stress conditions [19,20]. Proteins responsive to abscisic acid (ABA) were upregulated in drought-stressed seedling leaves, along with proteins associated with stress responses, and other metabolic pathways [21,22]. Switchgrass seedling root proteomes evaluated using tandem mass tags mass spectrometry (TMT-MS) analysis showed significant changes in plants experiencing drought and aluminum stress [22]. Taken together, these studies provide data on the utility of proteomics to investigate switchgrass physiology. In this study, we used label-free quantitative proteomics to evaluate temporal changes in protein abundances in rust-infected switchgrass cultivar Summer that is susceptible to Pn.

## 2. Results

### 2.1. Disease Progression and Change in Fungal Loads

**Figure 1:**
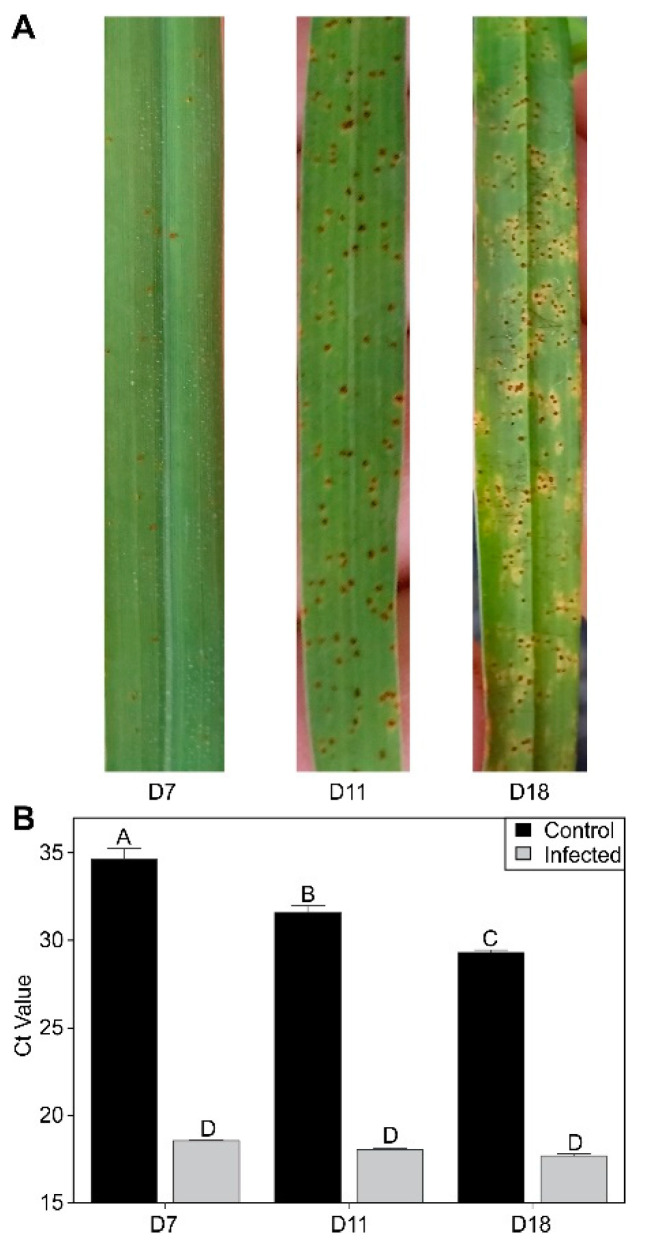
Disease progression. (**A**) Representative pictures of fungal development across the time course of the experiment. (**B**) Quantification of fungal DNA loads by qPCR in control and infected plants. Letters over each bar in Panel B denote statistical significance.

Infection of switchgrass by rust progressed over the time course of the experiment, with the appearance of a few pustules already evident by 7 DAI (Figure 1A).

Pustules were well differentiated by 11 DAI, and leaf damage was visible by 18 DAI (Figure 1A). Detection of fungal DNA loads was performed with qPCR and indicated little change in apparent fungal load between 7 and 18 DAI (Figure 1B). Some limited secondary infection of control plants was observed between 7 and 18 DAI, although the detected fungal loads in control plants were significantly lower in comparison to the infected plants. These infections likely arose from the proximity of plants within the greenhouse.

### 2.2. Total Protein Identified and Differentially Abundant

Collected MS2 spectra and resulting peptide spectrums were matched at a 1% false discovery rate (FDR). These data resulted in the identification of a total of 3862 proteins with high confidence. Of the 3862 proteins identified and quantified, 3632 were mapped to the switchgrass proteome v5.1 (https://phytozome-next.jgi.doe.gov/ (accessed on 23 February 2021), and 198 proteins were mapped to different *Puccinia* proteomes (Supplementary Materials). Across all comparisons, 1825 differentially accumulated proteins (DAPs) were identified. DAPs were further classified as enriched (EDAP) or diminished (DDAP) as a result of fungal infection.

### 2.3. Rust Infection Substantially Altered Host Proteomes

Rust infection caused significant changes to the switchgrass proteome and the numbers of DAPs increased substantially by 18 DAI. At 7 DAI, a total of 436 DAPs were found, 151 of which were significantly enriched and 285 proteins were significantly diminished in abundance in infected plants relative to control non-infected plants.

**Figure 2:**
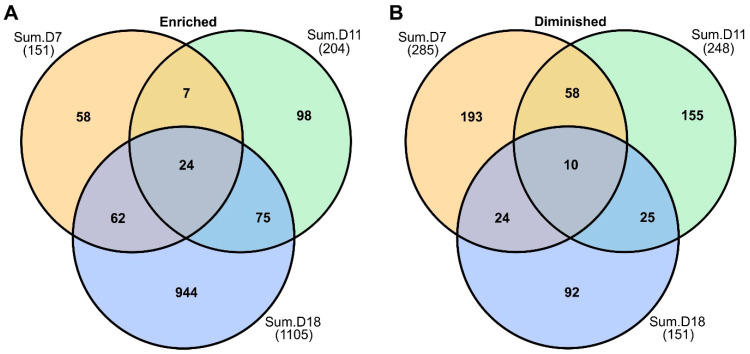
Venn diagram of enriched and diminished differentially abundant proteins (DAPs). (**A**) Shared and unique DAPs found in rust-infected Summer plants at 7, 11, and 18 DAI. (**B**) Shared and unique DDAPs found at 7, 11, and 18 DAI in Summer plants. See text for more details.

At 11 DAI, there was a total of 204 EDAPs and 248 DDAPs in infected plants relative to non-infected control plants. EDAPs increased to 1105 proteins, and 151 DDAPs were found at 18 DAI (Figure 2A,B). EDAPs increased with progression of disease, and DDAPs decreased with time. A total of 24 EDAPs were found to be similar across sampling dates (Figure 2A). Several of these proteins were associated with defense, such as PR proteins, chitinase, β-1-3-glucanase, and NAD(P)-linked oxidoreductases (Supplementary Materials). Among the 10 DDAPs found at all sampling dates in infected Summer plants, several were also associated with plastid functions. These included homologs of proteins required for photosystem biogenesis, photomorphogenesis, and directional growth.

### 2.4. STRING Analysis Using Arabidopsis Homologs and Annotations

A STRING analysis was performed for each sampling date using homology analysis. Switchgrass DAPs and their predicted Arabidopsis homologs were first identified from the genome annotation files. These predicted Arabidopsis homologs were next uploaded to STRING for network analysis (see methods). However, these extensive datasets accompanying many Arabidopsis proteins have not been directly referenced due to a lack of space, but the authors acknowledge the important contributions of these previous scientists. Readers can use the Arabidopsis annotations shown in the figures, or more completely in the Supplemental Information, to look at functions and primary references associated with each Arabidopsis entry. Unfortunately, few switchgrass proteins/genes have had their function(s) validated in-planta, limiting analysis to inference based primarily on their Arabidopsis homologs. One caveat in this analysis was the cross annotation of one Arabidopsis protein to multiple switchgrass proteins (diploid versus tetraploid genomes, gene duplication, and multiple loci encoding similarly annotated proteins with potentially different functions and roles). Moreover, this was not an issue for most of the predicted STRING clusters; there were occasions when switchgrass proteins with the same predicted Arabidopsis annotation were part of both the EDAP or DDAP clusters (see Section 4.5 and Supplementary Materials). Despite these occasional overlaps, STRING analyses revealed greater details of how the switchgrass proteome was modulated over time by rust infection.

### 2.5. Ribosomal Proteins and mRNA Splicing Were among EDAPs at 7 DAI

**Figure 3:**
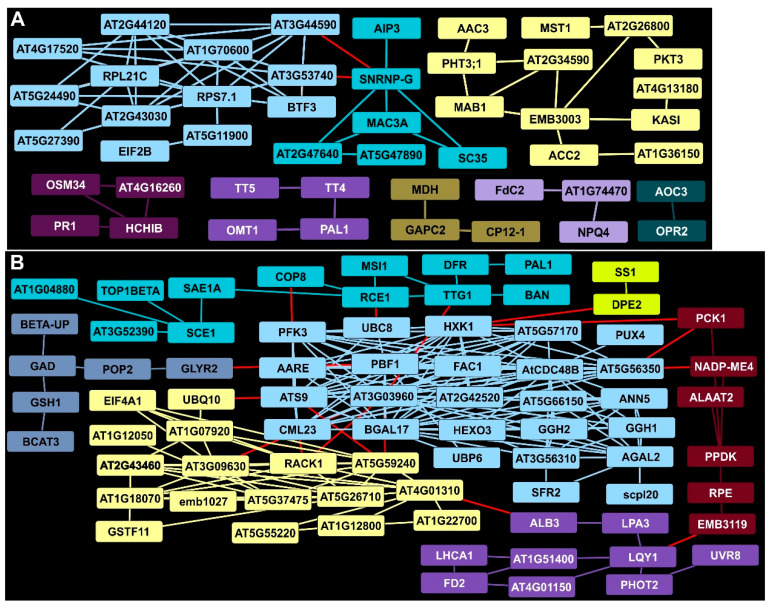
STRING analysis of DAPs in rust-infected Summer plants 7 DAI. (**A**) Clusters and connectivity of EDAPs. (**B**) Clusters and connectivity of DDAPs. Clusters containing Arabidopsis proteins with known interactions in databases are in boxes of the same color. Red lines indicate potential association with other clusters. Arabidopsis functional protein annotations are from the NCBI database. Switchgrass orthologs of Arabidopsis proteins are given in Supplementary Materials.

EDAP clusters at 7 DAI included one cluster containing several ribosomal proteins (light blue, Figure 3A) that was plausibly linked via an mRNA splicing factor (SNRNP-G) to a second cluster (aquamarine) that contained a switchgrass MAC3A ortholog. MAC3A was implicated in Arabidopsis innate immunity, suggesting that the accumulation of specific proteins was involved in defense. Several other proteins, enriched at 7 DAI in rust-infected plants, were directly linked to plant defense and thus could be components of switchgrass innate immunity. As examples, the basic chitinase (HCHIB, deep purple) was in a cluster with three other proteins, (PR1, OSM34, and AT4G16260), all with known roles in plant defense. Systemically acquired resistance mediated by hormones likely involves HCHIB. Two proteins required for JA biosynthesis (AOC3 and OPR2, slate) were also enriched at 7 DAI, providing possible links to HCHIB. Similarly, proteins required for supplying phenylpropanoid intermediates (PAL, TT4, TT5, and OMT1, purple) were identified. Other EDAPs were associated with organelle functions, such as MAB1, FdC2, and GAPC2 (Figure 3A).

### 2.6. Identification of a Central Hub for DDAPs at 7 DAI in Rust-Infected Summer Plants

AT3G03960, a TCP-1/cpn60 chaperonin family protein, and hexokinase 1 (HXK1) homologs were significantly lowered in abundance by 7 DAI in infected Summer plants and had the many predicted associations with other DDAPs (light blue, Figure 3B). This central hub contained several proteins with divergent roles in plant metabolism, such as those linked to sugar metabolism (BGAL17, HEXO3, AGAL2, PFK3), proteolysis (UBP6, UBC8, AARE), membrane trafficking (ANN5), and mitochondria (FAC1). Associations of the proteins in this cluster suggested links to other clusters containing proteins involved in hormone signaling, ribosome function and protein biosynthesis (AT5G59240, AT2G43460, and EIF4A1 as examples, yellow, Figure 3B), starch and sugar metabolism (DPE2, SS1, gold), chloroplast carbon fixation (PCK1, PPDK, dark red), and plastid function (LQY1, ALB3, PHOT2, LHCA1, purple). In addition, a cluster of proteins (slate, Figure 3B) was linked to amino acid metabolism (GAD, BCAT3,), synthesis of glutathione (GSH1), and glyoxylate reduction (GLYR2), potentially recalibrating cellular redox. Notably, some proteins associated with anthocyanins were DDAPs (aquamarine), and these included TTG1, a transducin/WD40 repeat-like superfamily protein needed for expression of BAN. Both BAN and DFR, needed for anthocyanin biosynthesis, were diminished, suggesting that phenylpropanoids were diverted to the formation of other compounds.

### 2.7. Plant Defense and Lignin Biosynthetic Clusters Were Enriched by 11 DAI

**Figure 4:**
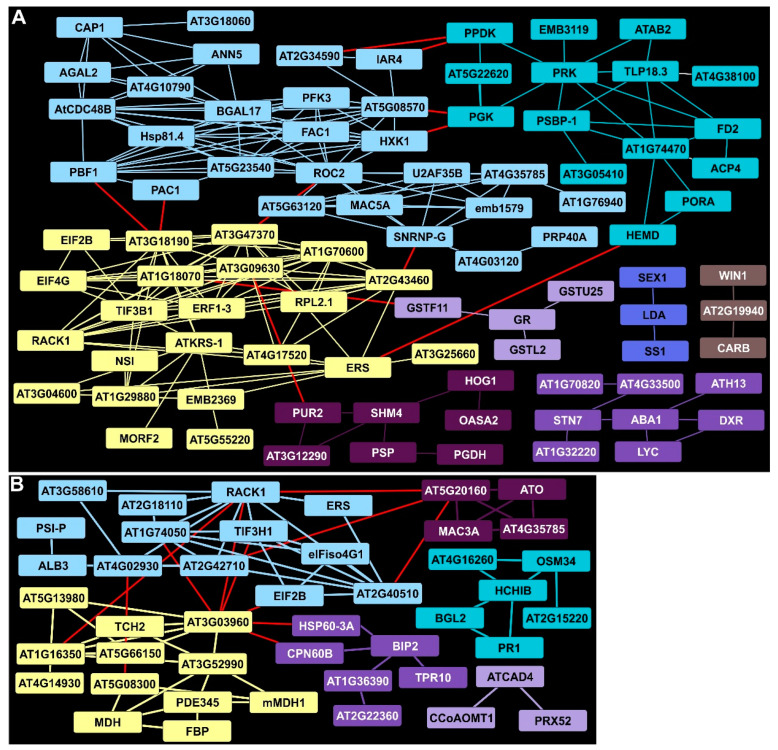
STRING analysis of DAPs in rust-infected Summer plants 11 DAI. (**A**) Clusters and connectivity of EDAPs. (**B**) Clusters and connectivity of DDAPs. Other details as given in Figure 3.

A cluster containing RACK1 detected at 7 DAI was still upregulated at 11 DAI and was connected to a larger group of proteins (blue, Figure 4A). RACK1 is involved in several hormone-dependent and growth responses in Arabidopsis. Other EDAPs associated with RACK1 included ribosomal proteins (AT1G74050, AT2G42710). This cluster also contained proteins of the synthesis machinery (such as TIF3H1, EIF2B, and eIFiso4G1). An aldolase-type TIM barrel family protein (AT1G16350, yellow, Figure 4A) interactor of RACK1 was found in a cluster containing several proteins involved in energy metabolism (such as MDH, FBP, and potentially another FBP-type enzyme, PDE345). TCH2, a calcium-binding protein, was found in a cluster that included cell wall-associated glycosyl hydrolases (AT5G13980 and AT5G66150). In Arabidopsis, TCH2 is implicated in nitric oxide (NO) generation as part of innate immunity signaling. Other EDAP clusters included one with several chaperones, (such as BIP2) suggestive of ER stress in rust-infected switchgrass plants (purple, Figure 4A). Lastly, a set of enzymes required for phenylpropanoid and or lignin biosynthesis (ATCAD4, CCoAOMT1 and PRX52) were enriched, suggesting that PAL induction at 7 DAI was likely linked to increased biosynthesis of phenylpropanoids (lilac, Figure 4A).

### 2.8. Protein Networks Indicated Continued Suppression of Primary Metabolism at 11 DAI

Continued suppression of primary metabolism was evident in the STRING clusters identified at 11 DAI in infected Summer leaves. There were, however, several changes in the proteins comprising these clusters relative to those detected at 7 DAI. The HXK1 DDAP cluster (light blue, Figure 4B) at 11 DAI contained 16 proteins not observed at 7 DAI and included several subunits of the 26S proteosome, RNA-binding proteins, and ROC2, a peptidyl-prolyl cis-trans isomerase activity suggesting increased proteolysis and protein misfolding. More evidence of downturns in metabolism were seen in a cluster of proteins linked to protein synthesis in different cellular compartments (yellow, Figure 4B). These included a cytosolic lysine-tRNA ligase (ATKRS-1) among other tRNA-ligases; elongation factors EIF2B, ERF1-3, and EIF4G, which are required for protein synthesis; and several ribosomal proteins including RPL2.1, a chloroplast ribosome component. Also, the significant reduction in levels of several proteins required for efficient plastid functions was consistent with the apparent downregulation of metabolism. On the carbon assimilation side, DDAPs included PPDK, PGK, and ACP4 (aquamarine). On the light perception side, the plastid kinase required for modulating thylakoid membrane state transitions, STN7, along with proteins associated with carotenoid biosynthesis, (ABA1, DXR, and LYC, purple, Figure 4B), starch metabolism (SEX1, LDA and SS1, blue), and plastid arginine biosynthesis (WIN1, an N-Acetyl-gamma-glutamyl-phosphate reductase, and CARB, brown), was present. Similarly, proteins needed for serine (OASA2, SHM4, and PSP) and purine biosynthesis (PUR2, AT3G12290) were reduced in levels (dark purple, Figure 4B).

### 2.9. Extensive Remodeling of the Proteome Occurred at 18 DAI

**Figure 5:**
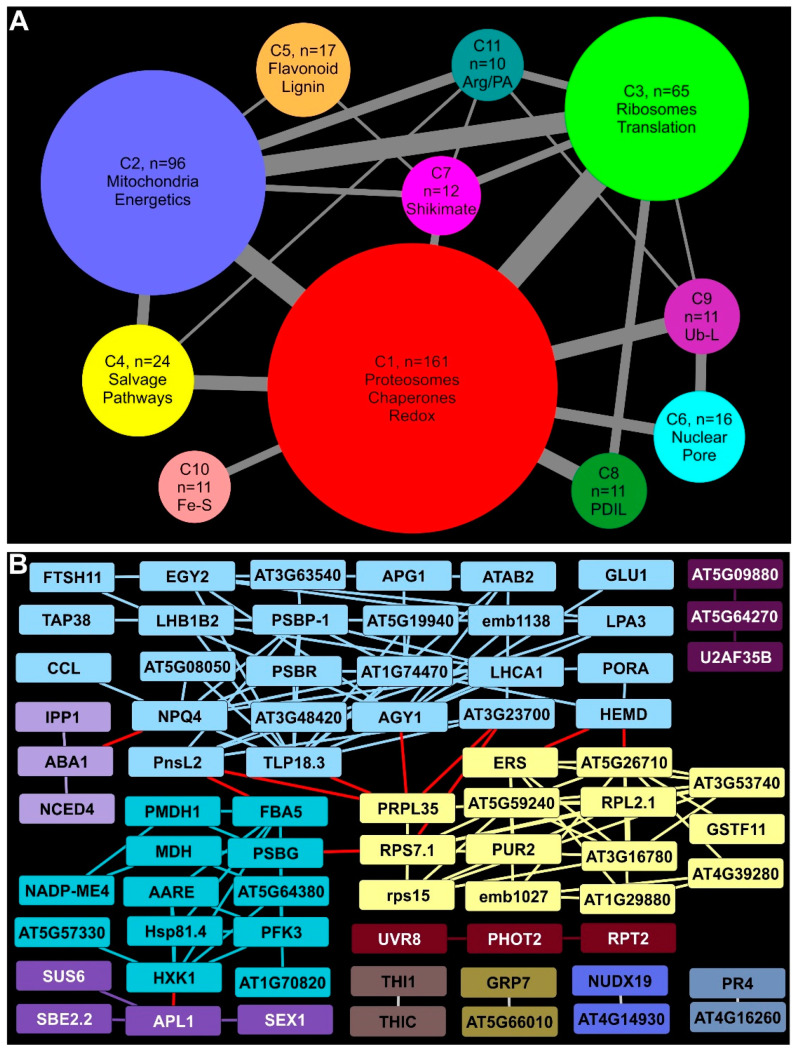
STRING analysis of DDAPs in rust-infected Summer plants 18 DAI. (**A**) Visual summary of the largest clusters and inter-cluster connectivity of EDAPs. Node size is proportional to the number of EDAPs in the cluster. Edge thickness is proportional to the number of edges connecting EDAPs in different clusters. (**B**) Clusters and connectivity of DDAPs. Other details as given in Figure 3.

Compared to earlier timepoints, there was a robust increase in the number of proteins with significant enrichment at 18 DAI (Figure 5A; Supplementary Materials). Among these EDAPs, 681 proteins were found to have Arabidopsis orthologs and were used for STRING analyses. The large number of EDAPs resulted in the identification of 53 clusters, most with fewer than 10 proteins (Supplementary Materials). Eleven clusters with 10 or more proteins were queried for functional annotations and represented as circles proportional to the number of proteins present (Figure 5A).

### 2.10. Proteosomal, Chaperone, and Redox Proteins Accumulated under Prolonged Rust Infection

The largest STRING-predicted cluster at 18 DAI (C1) contained 161 EDAPs broadly associated with redox mitigation, proteosomes, and chaperones, providing a glimpse into the reorganization of the switchgrass proteome in response to rust infection. Several proteosomal proteins, including PAD2, PAC1, FUS5, and AT5G23540, were increased in abundance at 18 DAI in rust-infected plants (red, Figure 5A; Supplementary Materials), suggesting the possibility of greater turnover of proteins in infected plants relative to non-infected controls. Notably, three prefoldins (PFDs) were also part of this cluster. PFDs are needed for folding actins and tubulins and can influence HSP70 binding to components of the spliceosome core. Levels of several switchgrass orthologs of Arabidopsis ribosomal proteins, translation elongation factors and proteins associated with mRNA biogenesis and stabilization were EDAPs, which signifies consistency with the previously mentioned finding. Switchgrass orthologs of eIFiso4G1 and PAB8 that play a role during viral infection in Arabidopsis were included in this cluster. Enrichment of redox-related proteins, such as catalases, glutaredoxins, thioredoxins, glutathione-S transferases (GSTs), monodehydroascorbate reductases, peroxidases, and a germin-like proteins was observed (Supplementary Materials). As these proteins localize to different cellular compartments, including the apoplast, a broad change in metabolism of reactive oxygen species (ROS) was indicated. Combined with apparent loss in plastid functions, these changes suggested an increasing need to balance cellular redox.

### 2.11. Mitochondria and Energy Metabolism Were Distinctly Affected in Infected Switchgrass Plants

Mitochondria and energy metabolism were significantly impacted with broad changes in enzymes involved in carbon utilization and energy transduction across different cellular compartments found in cluster C2. Other EDAPS in C2 included several proteins required for different aspects of mitochondrial C metabolism for energy (such as MAB1, SDH2-2, several pyruvate dehydrogenase subunits, and proteins of the ETS, blue, Figure 5A; Supplementary Materials) indicating that infection-related stress had modified mitochondrial energetics. Mitochondrial porins (VDAC1 and VDAC4) potentially induced by the pathogen were also enriched, as was FDH, which is accumulated in response to cellular stress and has a role in disease resistance. Cytosolic proteins mostly involved in sugar metabolism include GAPC1, TPI, LOS2, and AT3G02360 (annotated as a decarboxylating 6-phosphonoglucanate dehydrogenase, indicating substrate level generation of ATP and NADPH). A switchgrass homolog to Arabidopsis cytosolic GLN1-1 suggested to regulate GLN levels in Arabidopsis cells was also part of this cluster.

### 2.12. Ribosomal and Translation-Related Proteins Enrichment Indicated Substantial Protein Synthesis in Infected Plants

Cluster C3 (light green; Figure 5A) contained 65 switchgrass proteins, with a majority annotated as homologous to Arabidopsis ribosomal proteins, protein translation, and those needed for chaperone activity. These proteins were targeted to several cellular compartments, including the cytoplasm, mitochondria, and chloroplasts (Supplementary Materials). Notably, several proteins such as TIM8, TIM9, and TIM13 orthologs were enriched. TIM proteins act as chaperones for the import and insertion of imported proteins into mitochondrial membranes that strengthen data on the changes in proteins needed for mitochondrial functions impacted over time by rust infection (see above). Such as the enrichment of ribosomal proteins and those needed for ribosomal assembly and biogenesis, several protein translation initiation factors were also enriched (Supplementary Materials). Notably, some of the Arabidopsis orthologs used for the enriched switchgrass protein translation initiation factors have been linked to specific cellular functions. For example, AT2G46290 and EIF3G1 are needed for translation of mRNAs associated with cell proliferation, eIFiso4G1 has a role in defense, and AT2G24060 is implicated in leaf and chloroplast development (Supplementary Materials). Together with many enriched protein chaperones in this cluster, it may indicate a realignment of leaf metabolism to engender growth as a potential means to escape stress imposed by rust infection.

### 2.13. Proteins Needed for Salvage Pathways Were Enriched

Rust-imposed stress also resulted in the enrichment of many proteins involved in salvage pathways for both N and C that were found in cluster C4 (yellow, Figure 5A). Arabidopsis orthologs of switchgrass proteins enriched in this cluster included ADSS, a chloroplast-localized adenylosuccinate synthetase implicated in the salvage pathway in purine nucleotide metabolism; PYD1, a chloroplastic enzyme involved in pyramidine base catabolism; BETA-UP, required for recycling of N from nucleobases; enzymes needed for glyoxylate detoxification and maintenance of redox balance (GLYR2); and several others (Supplementary Materials).

### 2.14. Phenylpropanoid, Flavonoid, and Anthocyanin Biosynthetic Enzymes Were Enriched at 18 DAI

A hallmark of plant defense responses is the upregulation of the phenylpropanoid and downstream pathways utilizing cinnamic acid derivatives. Proteomic data and STRING analysis provided evidence for a broad enrichment of these interconnected pathways. Several proteins including PAL1, CCoAOMT1, ATCAD4, PRX52, and ALDH2C4 needed for phenylpropanoid/lignin biosynthesis were EDAPs, as were the following key enzymes required for flavonoid and anthocyanin biosynthesis: CHS (TT4), CHI (TT5), F3H, DFR, CHIL, and FLS1 (orange, Figure 5A; Supplementary Materials).

### 2.15. Other Notable Clusters

Six other clusters containing eleven to sixteen proteins were found during STRING analysis (Figure 5A). These included C6 (light blue) that contained several proteins, such as EMB2766, NUP1, and IMPA-1, linked to nuclear pores and transport across the nuclear membranes in Arabidopsis (Supplementary Materials). Cluster C7 (pink, Figure 5A) contained several proteins linked to shikimate and amino acid metabolism, including SKL1, SKL2, and a 3-hydroquinate synthase ortholog. A phospho-2-dehydro-3-deoxyheptonate aldolase and ADT2 needed for aromatic amino acid metabolism were also part of C7. Protein signatures for ER stress and changes in plastid metabolism were found in C8 (dark green, Figure 5A, Supplementary Materials). These included protein disulfide isomerases associated with ER stress (PDIL1-1, PDIL2-1, UNE5), heat shock proteins (BIP2, HSP60), and other protein chaperones (AT1G36390, CRT3), indicating a possible need to refold unfolded proteins and to combat stress. Clusters C9 (purple), C10 (salmon pink) and C11 (slate) contained proteins linked to ubiquitin ligases, those needed for sulfur and Fe-S metabolism, and arginine/polyamine metabolism, respectively (Supplementary Materials).

### 2.16. Plastid Biosynthetic Pathways Were Negatively Impacted by Rust Infection at 18 DAI

Many of the DDAPs in infected plants at 18 DAI (Figure 5B) were also found at lower abundances at 11 DAI (see Figure 4B), and included a large cluster of proteins linked to chloroplast metabolism (such as PORA, HEMD, LHCA1, and PSBP-1; light blue; Figure 5B). Proteins not detected with STRING analysis at 11 DAI were included in this cluster, including PSBR, NPQ4, and APG1, which are involved in plastoquinone biosynthesis, and TAP38, a phosphatase controlling state transitions. Together, these data indicate a continued loss of chloroplast function with time in rust-infected plants. Several proteins associated with HXK1 (aquamarine, Figure 5B) involved in primary metabolism included MDH, PFK3, NADP-ME4, and PMDH1. Smaller clusters linked to starch metabolism (purple), chloroplast thiazole biosynthesis (THI1, THIC; brown), and phototropin-linked genes (UVR8, PHOT2, and RPT2, maroon) were among the DDAPs. Switchgrass orthologs of Arabidopsis proteins associated with host defense (GRP7, gold; AT4G16260, slate) were diminished in abundance.

### 2.17. Changes in the Fungal Proteome

A total of 198 fungal proteins were identified in the datasets. Protein presence and abundances changed over time, with 148, 178, and 193 fungal proteins detected in leaf samples from infected Summer plants at 7, 11, and 18 DAI, respectively (Supplementary Materials). One protein, annotated as a 40S ribosomal protein, was found only in the 7 DAI leaves. Similarly, an uncharacterized protein was unique to the 11 DAI Summer leaves. Leaves from control plants had 44 proteins ascribed to fungus at 7 DAI, although no visible signs of infection were noted. For the most part, the statistical comparisons between infected versus control samples for fungal protein abundances were highly significant at each sampling date, indicating that most fungal proteins found were greatly enriched in the samples collected from infected plants. Most of the fungal proteins appeared to be associated with primary metabolic processes of the pathogen.

## 3. Discussion

Proteomic studies using switchgrass are relatively limited [19,20,21,22,23] but can bolster more extensive evaluation of the physiological and genetic responses of this crop to pathogens that impact yield and quality. The proteomic data in this study broadly support earlier observations [6,13,24,25,26,27,28,29,30] on the responses of Summer plants to biotic stressors while providing greater depth to the changes in cellular metabolism occurring in switchgrass in response to prolonged stress caused by Pn.

Genomes of rust fungi encode an extensive collection of effector proteins, and Pn is no exception [31,32]. Nandety et al. [32] found a total of 1031 putative secreted proteins in the Pn genome, and these included several classes of hydrolases and transport proteins. There were several Pn peptidases and dehydrogenases detected within the proteomics dataset, along with several kinases, although many appear to be linked to metabolic functions required for fungal growth and development (Supplementary Materials). In addition to these secretory proteins, Nandety et al. [32] identified 24 effector proteins that were common to the *Puccinia* genomes analyzed. Indeed, several of these potential effectors were found in the present proteomics datasets, and included superoxide dismutase, peroxidase, ATPase, NADH dehydrogenase, and an alpha galactosidase, lending support to the genomic predictions. Similarly, of the 11 pathogenicity genes identified by Nandety et al. [32] in Pn, protein products for the following 2 of these 11 genes were found: superoxide dismutase and peptidyl-prolyl cis-trans isomerase.

STRING analyses of the DDAPs at 7 DAI were characterized by significant changes to the cytosolic and organellar proteomes with a suppression of growth-related functions and an apparent upshift in metabolism to produce defense-related and stress-mitigating proteins. For the most part, the DDAPs at subsequent sampling dates indicated a continued reduction in primary assimilation of carbon as seen by the lowered abundances in proteins needed for chloroplast health, involved in carbon assimilation (PPDK), and involved in sugar metabolism (HXK1). Likewise, the DDAPs included the switchgrass STN7 kinase and the TAP34 phosphatase orthologs, suggesting loss of light harvesting efficiency and potentially the ability to deal with deleterious effects of excess light compounded by the loss of proteins required for protective pigment biosynthesis. Indirectly, the large number of EDAPs related to redox/stress mitigation further implicate greater ROS accumulation. Chloroplasts are major sources of cellular ROS [33], and excess ROS originating from impaired chloroplasts could add a significant energy burden for stress mitigation in rust-infected Summer plants. Plastid function was also impacted in Kanlow plants, although apparently not to the same extent as in Summer plants, and, in marked contrast, both PPDK and HXK1 were part of the EDAPs, which was potentially indicative of more normal cellular functions in infected Kanlow plants.

Rust effectors can specifically target chloroplasts [34,35,36] and influence plastid function, and this appears to be the case also for the switchgrass-Pn pathosystem. Chloroplasts are central to sensing plant stress, and changes in their metabolism affect other aspects of plant growth and development, with ROS being a major contributor to these processes [33,37]. In this context, rust infection appeared to depress chloroplast functions and enhanced ROS mitigating and defense-related proteins, suggestive of commonalities in the immune response components and plausibly to Pn effectors targeting chloroplasts. Changes in plant proteomes following rust infection have been studied in other plant pathosystems [38,39,40,41,42,43,44]. In general, these studies indicated a suppression of primary metabolism and an enrichment of proteins linked to the defense and/or biosynthesis of secondary metabolites, such as phenylpropanoids. Additionally, other classes of proteins have been shown to have important roles in conditioning plant defense, including receptors, members of signaling cascades, transcription factors, stress-mitigating enzymes, and proteins that regulate hormone biosynthesis, especially those related to jasmonic acid and salicylic acid. In concordance to these other reports in the literature, switchgrass proteomes, especially under prolonged Pn pressure, were modified with a suppression of proteins linked to primary metabolism and an enrichment of proteins linked to defense, biosynthesis of secondary metabolites, and stress-mitigating pathways.

Overall, the proteomic data evidenced the complexity of switchgrasses’ response to rust at the proteomic level and provided clues to the metabolic adaptations occurring in Summer plants. Changes in the proteomes of infected Summer plants mirrored fungal loads estimated by relative levels of fungal DNA. Lastly, STRING analysis also implicated switchgrass TCH2, MAC3A, and RACK1 orthologs to have roles in immunity; although, how they specifically impact the differential responses of the two switchgrass cultivars used in this study to Pn remains to be elucidated.

## 4. Methods

### 4.1. Plant Materials

Switchgrass plants of an upland cultivar Summer were raised from seeds in containers, as described previously [27]. After seedling emergence and growth to the first true leaf stage, each container was thinned to contain two seeds. Plants at the 4-leaf stage were subjected to rust inoculations.

### 4.2. Rust Inoculation

Urediniospores (NE-isolate) from previously infected switchgrass plants were collected into gelatin capsules, dehydrated in a desiccator for 5–7 days, stored at −80 °C, and used as a source of inoculum. Gelatin capsules containing rust spores were taken from the freezer, placed into microcentrifuge tubes, and heat shocked at 45 °C for 10–15 min using a heating block. After heat shock, lids were opened on tubes containing spores, and the tubes were placed into a desiccator to rehydrate for approximately 3 h. Approximately 150 mg of hydrated urediniospores were suspended in 50 mL of sterile distilled water with a drop of Tween 20. Inoculum was mixed well by shaking. Plants were sprayed approximately 30 cm from the top, making sure that each inoculated switchgrass plant received an even coating of inoculum. Control plants were similarly sprayed with water containing Tween 20. Once sprayed, control and inoculated switchgrass plants were placed in a humidity chamber at 100% humidity for 48 h, with 12 h light/dark cycle. At 48 h, racks containing plants were put into tubs containing water and placed in a greenhouse maintained with an average day temperature of 25–26 °C. Tubs were replenished with water every other day and a general-purpose fertilizer (20-20-20 NPK) was added once a week. Natural lighting was supplemented with LED lighting during early mornings and evenings.

### 4.3. Sample Collection and Processing for Proteomics

At each sampling date, only the 4th leaf [27] from 3 biological replicates of 10 plants each from 5 randomly chosen containers was excised from control and inoculated trays, cut into approximately 3–5 cm pieces, placed in 50 mL polypropylene tubes and flash-frozen with liquid N_2_. To limit variations that might be introduced by sampling leaves of different aged plants, new replicates at the next sampling time for excision of the 4th leaf were chosen once the sampled ones were discarded. Leaf materials were subsequently ground to a fine powder using liquid N_2_-chilled mortars and pestles. All ground plant materials were stored at −80 °C until processed. Approximately 200 ± 5 mg of ground leaf tissue was used for protein extraction. Proteins were extracted and analyzed as described previously [23] but with the following alterations. Briefly, proteins were extracted using Tris-buffered phenol (pH 8.8). Proteins precipitated from phenol using several washes with acetone and methanol were dissolved in 8 M urea, 5 mM DTT, and 0.1 M Tris-HCl pH 7.6 containing 1X protease inhibitor (Roche cOmplete Protease Inhibitor Cocktail EDTA-free). Forty µg of reduced proteins was alkylated with 15 mM iodoacetamide. Samples were diluted to 2 M urea and digested with Lys-C (1:40 enzyme/substrate ratio) at 25 °C for 1 h, and then with trypsin at 1:16 enzyme/substrate ratio at 37 °C for 20 h. A further aliquot of trypsin was added, and digestion continued for a further 4 h. Digests were acidified to pH 3.0 with formic acid, desalted on 50 mg Sep-Pack C18 reverse phase SPE columns (Waters Corp, Milford, MA, USA), and lyophilized. Lyophilized peptide digests were resolubilized in 2% acetonitrile containing 0.1% formic acid and analyzed with LC-MS/MS on an RSLCnano system (ThermoFisher Scientific, Waltham, MA, USA) coupled to a Q-Exactive HF mass spectrometer (ThermoFisher Scientific). Separation was performed on a C18 nano column (ACQUITY UPLC M-class, Peptide CSH 130A, 1.7 µm 75 µm × 250 mm, Waters Corp, Milford, MA, USA) at 260 nL/min with a linear gradient from 5–31% for 120 min. Acquired mass spectra were analyzed in Proteome Discoverer 2.2 software (ThermoFisher Scientific) and searched against the common contaminants database cRAP (116 entries, www.theGPM.org (accessed on 1 January 2012)). the version 5.1 of the switchgrass genome (79,335 entries, www.phytozome-next.jgi.doe.org (accessed on 23 February 2023)), and a combined database of 3 UniProt reference proteome of Puccinia (*Puccinia graminis* f. sp Tritici, 15,688 entries; *Puccinia striiformis* f. sp. Tritici, 19,368 entries; *Puccinia sorghi*, 21,032 entries) using Mascot 2.6.2. Protein N-terminal acetylation, asparagine and glutamine deamidation, methylation, and methionine oxidation were set as variable modifications, whilst Cys carbamidomethylation was specified as a fixed modification. A maximum of two missed cleavages with trypsin were permitted and the precursor and fragment mass tolerances were set to 10 ppm and 0.02 Da, respectively. Peptides were validated using Percolator with a 0.01 posterior error probability (PEP) threshold. The data were searched using a decoy database to set the false discovery rate to 1% (high confidence).

### 4.4. Experimental Design and Statistical Analyses

The experimental design was a 2 × 3 factorial, with 2 treatments (control and inoculated) × 3 biological replicates of 5 containers (10 combined plants (genotypes) per replicate). For proteomic analyses, a total of 18 samples were processed from Summer control and infected plants (3 sampling times, 7, 11, 18 DAI × 3 biological replicates). Significance measures of proteins were analyzed using ANOVA, which provides adjusted *p*-values using the Benjamini–Hochberg method for all the calculated ratios, as previously described [23].

### 4.5. Functional Annotation and STRING Analysis

STRING analysis [45] was performed using the “Best Arabidopsis Hit” assignments from the switchgrass gene annotation file (Phytozome 13). Due to the nature of the best-hit assignments, non-homologous switchgrass loci may share the same Arabidopsis annotation (see Supplementary Materials). As a result, the same Arabidopsis gene may appear in competing/conflicting/opposing STRING gene sets. STRING networks were created using text mining, experiments, databases, and co-expression as active interaction sources with a “high confidence” minimum interaction score threshold. Each network was subdivided into clusters using MCL clustering with an inflation parameter of 1.4, as described for STRING analysis. All identified links were combined and weighted to give a score for the relationship between two proteins, with a value greater than 0.7 resulting in a line connecting the two proteins. Proteins in clusters of the same color were likely to be part of an interacting cluster and linked by lines of the same color; red lines connecting separate clusters suggest that existing data in Arabidopsis potentially links these clusters in some fashion. Within the predicted STRING clusters, proteins are identified by their Arabidopsis annotations, and functional descriptions associated with their Gene IDs in NCBI (we have not cited most of the primary literature associated with each Arabidopsis gene locus since they are readily available within NCBI. However, we acknowledge the seminal contributions of these authors). To make it easier to locate clusters/interactomes/clusters, only a few members are provided in the text accompanying each figure. A full list of proteins found in each cluster and predicted interactions (edges) within each cluster are provided in Supplementary Materials.

### 4.6. Estimation of Pn DNA Levels in Leaves

Relative fungal loads were estimated using DNA-based qRT-PCR. Briefly, DNA was purified from all harvested samples using a Mag-Bind Plant DNA Plus Kit (Omega Bio-Tek, M1128, Norcross, GA, USA) with a KingFisher Flex System (ThermoFisher Scientific). Ten ng of total DNA was used per well with the Pn (*Puccinia emaculata*) specific ITS primers, SGR-SP1-FW and SGR-SP1-RV, identified by Uppalapati et al. [16]. Duplicate reactions were run per sample on a CFX Connect Real-Time System (Bio-Rad, Hercules, CA, USA) using SsAdvanced Universal SYBR Green Supermix (Bio-Rad, 172-5271). Delta-Ct values were calculated between control (mock) samples and rust-infected samples at each time point.

All methods were performed in accordance with the relevant guidelines/regulations/legislations.

## Data Availability

The MS proteomics data have been deposited in PRIDE archive PRIDE—Proteomics Identification Database (ebi.ac.uk). Project accession: PXD036978. Reviewer account details: username: reviewer_pxd036978@ebi.ac.uk; password: xTClhV55. Project name: Dynamic Reconfiguration of Switchgrass Proteomes in Response to Rust (*Puccinia novopanici*) Infection.

## Supplementary Materials

The following supporting information can be downloaded at: https://www.mdpi.com/article/10.3390/ijms241914630/s1.

## Abbreviations

ABA, abscisic acid; DAI, days after infection; DAP, differentially accumulated protein; DDAP, diminished differentially accumulated protein; EDAP, enriched differentially accumulated protein; MEP, methylerythritol pathway; NLR, nucleotide-binding site leucine-rich repeat protein; NO, nitric oxide; Pn, *Puccinia novopanici*; cv, cultivar; ROS, reactive oxygen species.
